# [^177^Lu]Lu-PSMA-617 in Patients with Progressive PSMA+ mCRPC Treated With or Without Prior Taxane-Based Chemotherapy: A Phase 2, Open-Label, Single-Arm Trial in Japan †

**DOI:** 10.3390/cancers17142351

**Published:** 2025-07-15

**Authors:** Kouji Izumi, Ryuji Matsumoto, Yusuke Ito, Seiji Hoshi, Nobuaki Matsubara, Toshinari Yamasaki, Takashi Mizowaki, Atsushi Komaru, Satoshi Nomura, Toru Hattori, Hiroya Kambara, Shaheen Alanee, Makoto Hosono, Seigo Kinuya

**Affiliations:** 1 Department of Integrative Cancer Therapy and Urology, Graduate School of Medical Sciences, Kanazawa University, Kanazawa 920-8640, Japan; 2 Department of Renal and Genitourinary Surgery, Hokkaido University, Sapporo 060-8638, Japan; ryu_matsumoto_0317@yahoo.co.jp; 3 Department of Urology, Yokohama City University Graduate School of Medicine, Yokohama 236-0004, Japan; yitou1@yokohama-cu.ac.jp; 4 Department of Urology, Fukushima Medical University School of Medicine, Fukushima City 960-1295, Japan; hosiseiji@gmail.com; 5 Department of Medical Oncology, National Cancer Center Hospital East, Kashiwa 277-8577, Japan; nmatsuba@east.ncc.go.jp; 6 Department of Urology, Kobe City Medical Center General Hospital, Kobe 650-0047, Japan; toshinari_yamasaki@kcho.jp; 7 Department of Radiation Oncology and Image-Applied Therapy, Graduate School of Medicine, Kyoto University, Kyoto 606-8501, Japan; mizo@kuhp.kyoto-u.ac.jp; 8 Prostate Center and Division of Urology, Chiba Cancer Center, Chiba 260-0801, Japan; akomaru@chiba-cc.jp; 9 Novartis Pharma K.K., Tokyo 105-0001, Japan; satoshi.nomura@novartis.com (S.N.); toru.hattori@novartis.com (T.H.); hiroya.kambara@novartis.com (H.K.); 10 Senior Clinical Development, RLT Prostate, Novartis Pharmaceuticals Corporation, East Hanover, NJ 07936, USA; shaheen.alanee@novartis.com; 11 Department of Radiology, Kindai University, Osaka 577-8502, Japan; hosono@med.kindai.ac.jp; 12 Department of Nuclear Medicine, Kanazawa University Hospital, Kanazawa 920-8641, Japan; kinuya@med.kanazawa-u.ac.jp

**Keywords:** ^177^Lu-PSMA-617, ^68^Ga-PSMA-11, Radioligand therapy, Japan

## Abstract

**Background:** This Phase 2 trial evaluated the efficacy, tolerability, and safety of [^177^Lu]Lu-PSMA-617 (^177^Lu-PSMA-617) in patients with ≥1 measurable lesion and progressive prostate-specific membrane antigen-positive (PSMA+) metastatic castration-resistant prostate cancer (mCRPC) in Japan. **Methods:** This study comprises four parts; data from three parts are presented here. Part 1 evaluated safety and tolerability; Parts 2 (post-taxane) and 3 (pre-taxane/taxane-naive) assessed the overall response rate (ORR; primary endpoint), overall survival (OS), radiographic progression-free survival (rPFS), disease control rate (DCR), PFS, and safety; and Part 4 is the expansion part. Patients received 7.4 GBq (±10%) ^177^Lu-PSMA-617 Q6W for up to six cycles. **Results:** Of the 35 patients who underwent a [^68^Ga]Ga-PSMA-11 (^68^Ga-PSMA-11) PET/CT scan, 30 received ^177^Lu-PSMA-617 (post-taxane, n = 12; pre-taxane, n = 18). No dose-limiting toxicity was noted in Part 1 (n = 3). Post- and pre-taxane patients had a median of three and five cycles, respectively. The primary endpoint, ORR, met the pre-specified threshold, with the lower limit of the 90% confidence interval (CI) above the threshold of 5% for post-taxane and 12% for pre-taxane. Post- and pre-taxane patients had an ORR of 25.0% (90% CI: 7.2–52.7) and 33.3% (90% CI: 15.6–55.4), respectively. In post- and pre-taxane patients, the DCR was 91.7% and 83.3%, the median rPFS was 3.71 and 12.25 months, and the median PFS was 3.71 and 5.59 months, respectively. The median OS was 14.42 and 12.94 months in post- and pre-taxane patients, respectively. The most common adverse events were constipation, decreased appetite, decreased platelet count, anemia, and nausea. **Conclusions:** The primary endpoint (ORR) was met. The safety profile of ^177^Lu-PSMA-617 was consistent with the VISION and PSMAfore studies, with no new safety signals in the Japanese patients with mCRPC.

## Simple Summary

This summary provides results of a phase 2 clinical study from Japan, which tested the radioligand therapy, [^177^Lu]Lu-PSMA-617, among Japanese patients with metastatic castration-resistant prostate cancer (mCRPC) who have or have not received chemotherapy previously. In mCRPC, the prostate cancer spreads to other parts of the body and it continues to grow even when the testosterone levels in blood are decreased to lower values. Researchers studied how well [^177^Lu]Lu-PSMA-617 works by looking at whether it shrinks tumors, improves patients’ overall health, delays the cancer from getting worse, and what side effects it may cause. The study showed that [^177^Lu]Lu-PSMA-617 treatment has a favorable anti-cancer effect in mCRPC patients. The side effects include constipation, loss of appetite, low platelet counts, low red blood cells, and vomiting. This treatment could be a new therapy for managing mCRPC with manageable side effects.

## 1. Introduction

Patients with metastatic castration-resistant prostate cancer (mCRPC) have a poor prognosis characterized by limited survival and a marked deterioration in quality of life (QOL) due to the rapid progression of the disease [1,2,3,4]. Over the past decade, advancements in the understanding of the genomic landscape and molecular biology of mCRPC have informed the development of novel therapeutic strategies.

Several therapeutic agents have been approved for the treatment of mCRPC, with consensus guidance provided by major guidelines including those from the NCCN, ESMO, and EAU–EANM–ESTRO–ESUR–SIOG [5,6,7]. In Japan, similar agents, with the exception of Sipuleucel-T, have been approved for the treatment of mCRPC and are recommended by the Japanese Urological Association’s clinical practice guideline for prostate cancer (version 2023) [8]. Notably, there are no significant differences in mCRPC treatment approaches between Japan and the United States of America (USA) or other European Union (EU) countries.

Despite significant advancements in the treatment landscape of metastatic castration-resistant prostate cancer, current options remain suboptimal, as many are non-biomarker selected, associated with significant toxicity, or used in the early-stage disease, often resulting in cross-resistance [9,10]. Among patients previously treated with an androgen receptor pathway inhibitor (ARPI), several mechanisms have been implicated in the development of resistance, limiting the efficacy of a second ARPI [11,12,13,14]. On the other hand, many patients do not receive chemotherapy, primarily due to pre-existing medical conditions or its associated toxic effects [15]. This highlights the urgent need for innovative, well-tolerated therapies that can preserve QOL, improve clinical outcomes, serve as alternatives to sequential ARPIs, and delay the use of taxanes in this patient population [9,16].

Prostate-specific membrane antigen (PSMA) is a transmembrane protein highly expressed in all stages of prostate cancer and is an independent prognostic biomarker [17,18,19,20]. PSMA-targeted radioligand therapy has emerged as a promising treatment approach, selectively delivering cytotoxic radioactivity to PSMA-expressing lesions. This approach optimizes therapeutic efficacy by maximizing on-target effects while minimizing off-target toxicity, offering a unique biomarker-driven therapeutic strategy for patients with prostate cancer [21,22,23].

^177^Lu-PSMA-617 is a PSMA-targeted radioligand therapy that delivers DNA strand-breaking radiation that may lead to prostate cancer cell death [24]. The beta radiations facilitate “crossfire” irradiation of the surrounding cells, thus addressing the critical need for biomarker-guided therapy for advanced prostate cancer [25]. ^68^Ga-PSMA-11 positron emission tomography (PET)/computed tomography (CT) imaging enables identification of PSMA-positive lesions that can be targeted with ^177^Lu-PSMA-617 and has been used in Phase 2/3 studies to guide the selection of patients with PSMA-positive mCRPC suitable for ^177^Lu-PSMA-617 therapy [23,26,27,28].

Clinical evidence from two Phase 3 trials, VISION and PSMAfore, has demonstrated that ^177^Lu-PSMA-617 significantly improves radiographic progression-free survival (rPFS) with a favorable safety and tolerability profile in both pre- and post-taxane settings for PSMA-positive mCRPC patients previously treated with ARPIs [27,28]. In the VISION trial, ^177^Lu-PSMA-617 extended overall survival (OS) by 4 months and reduced the risk of radiographic progression or death by 60% compared to the standard of care (SoC) alone, while preserving the QOL [27]. As a result, ^177^Lu-PSMA-617 has been approved in the USA and the EU for the treatment of adult patients with PSMA-positive mCRPC who have received ARPIs and taxane-based chemotherapy [29,30]. In the PSMAfore trial, interim analysis showed that treatment with ^177^Lu-PSMA-617 resulted in a longer median PFS compared with a change in ARPI [28]. Both trials included limited representation of Japanese patients, highlighting the need for region-specific clinical data to inform treatment decisions in this population.

This prospective Phase 2 trial aimed to evaluate the efficacy, tolerability, safety, pharmacokinetics, and dosimetry of ^177^Lu-PSMA-617 in patients with progressive PSMA-positive mCRPC in Japan, targeting a population comparable to that of the Phase 3 VISION and PSMAfore studies.

## 2. Materials and Methods

### 2.1. Study Design

This is a prospective, open-label, multicenter, single-arm, Phase 2 trial (NCT05114746) of patients with progressive PSMA-positive mCRPC in Japan. This is a four-part study, comprising Part 1 (safety run-in part), Part 2 (post-taxane part), Part 3 (pre-taxane part), and Part 4 (expansion part). Dosimetry and pharmacokinetic assessments of ^68^Ga-PSMA-11 were optional, while those for ^177^Lu-PSMA-617 were mandatory in Part 1 and planned among 4 to 6 patients in selected sites. Patients enrolled in Part 1 were also considered in Part 2 or 3 for efficacy and safety assessment. Since Part 4 data are not included in the primary analysis, only Parts 1, 2, and 3 are described here. The study was conducted in compliance with the Good Clinical Practice (GCP) guidelines and the Declaration of Helsinki. The investigational products, ^177^Lu-PSMA-617 and PSMA-11, were provided by Novartis Pharma K.K. (Tokyo, Japan), and ^68^Ge/^68^Ga-Generator was provided by Eckert and Ziegler Radiopharma GmbH (Berlin, Germany).

### 2.2. Key Eligibility Criteria

Inclusion criteria for this study required patients with PSMA+ mCRPC who had at least one measurable lesion on computed tomography (CT) or magnetic resonance imaging (MRI) based on the Prostate Cancer Working Group 3 (PCWG3)-modified Response Evaluation Criteria in Solid Tumors (RECIST) v1.1 criteria in Parts 1, 2, and 3. Prior confirmation of prostate cancer and documented progressive mCRPC was necessary. A positive ^68^Ga-PSMA-11 PET/CT scan, as determined by central review (Parts 1, 2, and 3), was required before enrolling in the ^177^Lu-PSMA-617 treatment period. The presence of PSMA-positive lesions was defined as a ^68^Ga-PSMA-11 uptake greater than that of normal liver in one or more lesions of any size in any organ system. The presence of PSMA-negative lesions was defined as PSMA uptake equal to or lower than that of normal liver in any lymph node with a short axis of at least 2.5 cm, in any metastatic solid-organ lesions with a short axis of at least 1.0 cm, or in any metastatic bone. Castrate levels of testosterone (<50 ng/dL or <1.7 nmol/L) were allowed. The study included both post-taxane and pre-taxane populations. Post-taxane patients had Eastern Cooperative Oncology Group performance status (ECOG PS) 0–2, prior treatment with ≥1 ARPI, and prior treatment with 1 to 2 prior taxane regimens.

The pre-taxane group included patients who had received one ARPI in either hormone-sensitive prostate cancer or CRPC setting, had documented progression on therapy, were considered appropriate for delaying taxane-based chemotherapy and candidates for treatment with an alternate ARPI, and had an ECOG PS of 0–1. Further details are available in the Supplementary Materials.

### 2.3. Study Endpoints

The primary endpoint was to evaluate the dose-limiting toxicity of ^177^Lu-PSMA-617 during cycle 1 (6 weeks) in Part 1 and confirm the overall response rate (ORR) as per PCWG3-modified RECIST v1.1 criteria based on a local review before the initiation of a new anticancer treatment in Part 2 and 3. The secondary endpoints included OS, rPFS, PFS, ORR based on central review, disease control rate (DCR), duration of response (DOR), time to a symptomatic skeletal event, biochemical response (prostate-specific antigen [PSA], alkaline phosphatase [ALP], and lactate dehydrogenase [LDH] levels), health-related quality of life (HRQOL), and safety. Pharmacokinetics and dosimetry of ^68^Ga-PSMA-11 and ^177^Lu-PSMA-617 have been evaluated and published elsewhere.

### 2.4. Treatment

Patients in the pre-taxane population received intravenous injections of ^177^Lu-PSMA-617 every 6 weeks (±1 week) at a dose of 7.4 GBq (±10%) for up to 6 cycles. Completion of the study treatment in this group was defined as the administration of all 6 planned cycles of ^177^Lu-PSMA-617. Patients in the post-taxane population received a combination with SoC. ^68^Ga-PSMA-11 was administered intravenously at screening to assess PSMA positivity using a PET/CT scan. If ^177^Lu-PSMA-617 treatment was discontinued due to an adverse event (AE) or an abnormal lab value for the post-taxane population, SoC could be continued per the local investigator’s discretion until disease progression based on PCWG3-modified RECIST v1.1 for soft tissue lesions. Completion of the study treatment in the post-taxane population was investigator-dependent with no pre-determined cycle number for SoC.

### 2.5. Assessments

Efficacy assessments followed the PCWG3-modified RECIST 1.1 criteria, focusing on organ, soft tissue, and nodal lesions, and the PCWG3 criteria for bone disease. For the primary endpoint (ORR) analysis, a responder was defined as a patient with a response (complete response [CR] or partial response [PR]) per the PCWG3-modified RECIST v1.1, confirmed by a second consecutive tumor assessment ≥ 4 weeks later. ORR was defined as the proportion of responders among patients with measurable disease at baseline in the primary analysis. Additionally, tumor shrinkage was evaluated as an exploratory endpoint. Tumor shrinkage was defined as the proportion of patients with the best percentage change from baseline in the sum of diameters of target lesions, among those with measurable disease at baseline. Radiographic imaging included a baseline CT/MRI of the chest, abdomen, and pelvis and a bone scan to record sites of bone disease. Further imaging was conducted every 8 weeks for the first 24 weeks and every 12 weeks thereafter until disease progression was detected. Clinical progression was determined by cancer-related pain escalation, a need for new therapy, or deterioration in ECOG PS to ≥3. PSA response was monitored until disease progression was detected. ALP and LDH were assessed for biochemical response and safety. Time to first symptomatic skeletal event was measured for bone fractures, spinal cord compression, orthopedic intervention, or requirement for radiation to relieve bone pain. Pain score and HRQOL were assessed periodically. Tolerability and safety assessments included monitoring of Dose Limiting Toxicity (DLT) during cycle 1 and AEs and severe AEs for ^68^Ga-PSMA-11 and ^177^Lu-PSMA-617, including physical examination, vital signs, lab evaluations, electrocardiogram (ECG), and concomitant medications.

### 2.6. Statistical Analysis

The primary analysis included patients from Parts 1 to 3 of the study. Statistical Analysis System version 9.4 or later was used to perform all data analyses. The full analysis set included all patients who received at least one dose of ^68^Ga-PSMA-11, regardless of administration of ^177^Lu-PSMA-617. The primary analysis set was the primary population for efficacy data and included all the patients who were PSMA+ and received at least one dose of ^177^Lu-PSMA-617. The efficacy analysis for ^177^Lu-PSMA-617 was evaluated for the post-taxane and pre-taxane populations, respectively. For the primary analyses, the ORR was compared with the threshold on patients with measurable disease at baseline in the primary analysis set, using an exact binomial test at a one-sided α level of 0.05. The thresholds were 5% and 12% for the post-taxane population and the pre-taxane population, respectively, and a treatment effect was to be concluded if the lower bound of the two-sided 90% CI of the ORR > the threshold. The threshold of 5% for the post-taxane population reflects a third-line or later setting for patients with mCRPC who had previously received at least one ARPI and two taxane regimens, or one taxane regimen if deemed unsuitable for a second. This population has no established standard therapies, and limited evidence supports existing options. In this context, an ORR exceeding 5% is considered clinically meaningful.

For the pre-taxane population, the 12% threshold was informed by ORR data from recent phase 3 trials (CARD, PROfound, and IMbassador250), evaluating second-line ARPIs in patients with mCRPC who had progressed on prior ARPI or ARPI plus taxane therapy. The reported ORRs in the control arms of these studies were 11.5%, 4.0%, and 7.4%, respectively [12,13,31]. Based on these findings, an ORR exceeding 12% was prespecified as a clinically meaningful benchmark for patients in whom deferral of taxane-based chemotherapy is considered appropriate. The study aimed to enroll a target sample size of 12 patients for the post-taxane group and 16 for pre-taxane group based on statistical power calculations. Further details of the statistical analysis are available in the Supplementary Materials.

## 3. Results

### 3.1. Evaluation of Tolerability (Safety Run-In Part, Part-1)

No DLTs were reported. In the absence of DLTs among the first three patients enrolled in Part 1 to assess the tolerability of ^177^Lu-PSMA-617, along with other available safety data, the tolerability of ^177^Lu-PSMA-617 at 7.4 GBq in Japanese patients was confirmed. Consequently, screening/enrolment for Part 2 and Part 3 was initiated.

### 3.2. Study Population

**Figure 1:**
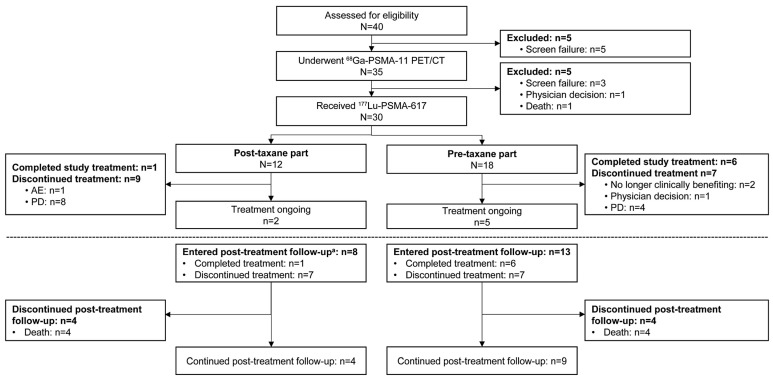
Patient disposition. A total of 40 patients were screened; 35 underwent ^68^Ga-PSMA-11 PET/CT. Ten were excluded before treatment (eight screen failures, one physician decision, one pre-treatment death). A total of 30 patients received ^177^Lu-PSMA-617: 12 post-taxane and 18 pre-taxane. In the post-taxane group, one completed treatment, nine discontinued (eight PD, one AE), and two remained on treatment. In the pre-taxane group, six completed, seven discontinued (four PD, two no clinical benefit, one physician decision), and five remained on treatment. Twenty-one patients entered follow-up; eight deaths occurred. ^a ^Two patients did not enter post-treatment follow-up. In the post-taxane population, completion of the study treatment was defined as completion of both [^177^Lu]Lu-PSMA-617 and SoC. Although 5 patients (5 of 12, 41.7%) completed all six infusions of [^177^Lu]Lu-PSMA-617, only 1 patient had also completed SoC at the time of data cut-off. Therefore, only one patient was considered to have completed full study treatment. In the pre-taxane population, completion of study treatment was defined as completion of six cycles of [^177^Lu]Lu-PSMA-617 along with the associated follow-up. Among the 7 patients (7 of 18; 38.9%) who completed all six infusions, 1 was still undergoing follow-up for the sixth cycle at the time of data cut-off. Therefore, six patients were considered to have completed study treatment. ^177^Lu, lutetium-177; ^68^Ga, gallium-68; AE, adverse event; SoC, standard of care; CT, computed tomography; PD, progressive disease; PET, positron emission tomography; PSMA, prostate-specific membrane antigen.

Overall, 40 patients were screened, of which 5 were excluded due to screen failure. Among the 35 patients who underwent a ^68^Ga-PSMA-11 PET/CT scan, 30 patients (post-taxane, n = 12; pre-taxane, n = 18) received ^177^Lu-PSMA-617 treatment and 5 were not treated due to screen failure (n = 3), physician’s decision (n = 1), and patient death (n = 1) (Figure 1). Of the 35 patients administered with ^68^Ga-PSMA-11, 33 met the PSMA PET eligibility criteria and 2 did not meet the criteria (one had no PSMA+ lesions, and the other had a PSMA-negative solid organ lesion with a short axis ≥ 1.0 cm).

In the safety analysis set (SAS), ^68^Ga-PSMA-11 was administered at a median (range) dose intensity of 160.00 MBq (112.8–243.0). The median (range) duration of exposure to ^177^Lu-PSMA-617 was 4.17 months (2.8–9.5) and 6.82 months (2.8–9.2) in the post- and pre-taxane population, respectively. The median (25th to 75th percentile) cumulative dose was 22.50 GBq (20.76–43.34) and 36.08 GBq (28.12–43.38) in the post- and pre-taxane setting, respectively. Overall, 41.7% of the post-taxane population and 38.9% of the pre-taxane population received six infusions of ^177^Lu-PSMA-617. A median (range) of 3 (2–6) injections were administered in the post-taxane population and 5 (2–6) injections in the pre-taxane population.

At the data cut-off date (8 December 2023), of the 30 patients who received ^177^Lu-PSMA-617, 7 (23.3%) completed the study treatment [1 post-taxane and 6 pre-taxane], 7 (23.3%) were on treatment and the remaining 16 (53.3%) discontinued the treatment, primarily due to progressive disease (PD; n = 12; 40%). Of these, 8 patients were in the post-taxane population (8/12 patients, 66.7%) and 4 in the pre-taxane population (4/18 patients, 22.2%); (Figure 1). Among 21 patients who entered the post-treatment follow-up, 8 patients died.

The demographic and disease characteristics of the patients at baseline and their previous treatments are presented in Table 1. The patient population is balanced between the two arms, with no major differences compared to the pivotal trial VISION for the post-taxane group and PSMAfore for pre-taxane group. In the post-taxane population, all patients had received prior taxane treatment with docetaxel, and 75.0% (9/12) had received cabazitaxel.

### 3.3. Primary Efficacy Results

**Figure 2:**
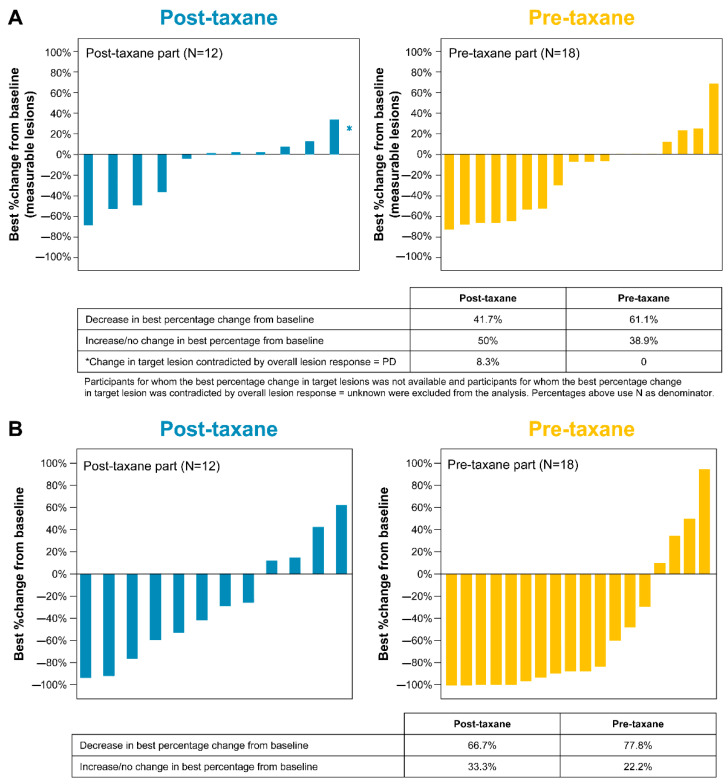
(**A**) Tumor response: best percentage change from baseline in sum of diameters (based on local review). (**B**) Biochemical response: best percentage change in PSA.

The study met the primary endpoint of achieving a clinically meaningful improvement in ORR based on local radiology review for both the post-taxane and pre-taxane populations. For the post-taxane population, ORR per local radiology review met the pre-specified threshold of primary endpoint, with the lower boundary of the 90% CI above the threshold of 5%. The ORR in this group was 25.0% (90% CI: 7.2–52.7), with PR noted in three patients (25.0%) and no CR. In the pre-taxane population, ORR also met the pre-specified threshold, with the lower boundary of the 90% CI above the threshold of 12%. The ORR in this group was 33.3% (90% CI: 15.6, 55.4), with CR in four patients (22.2%) and PR in two patients (11.1%) (Table 2). The DCR in patients with a measurable disease at baseline was 91.7% (95% CI: 61.5, 99.8) and 83.3% (95% CI: 58.6, 96.4) in the post- and pre-taxane population, respectively. Tumor shrinkage was observed in 41.7% and 61.11% of the post- and pre-taxane population, respectively (Figure 2A).

The overall concordance rate of BOR for soft tissue between the local and central radiology reviews for the post-taxane population was 25.0% and 66.7% in the pre-taxane population. This discordance may be the result of approximately half of the post-taxane participants being classified as not having measurable disease at baseline by central review. According to the RECIST criteria, only patients with measurable disease at baseline are eligible for ORR evaluation. As a result, their BOR was recorded as “Unknown” centrally, despite being evaluable by local review. Differences in lesion selection, particularly in cases involving small tumors or osteoblastic bone metastases, and the asymmetry of clinical context between local and central reviewers may have further contributed to the discordance. There was no significant difference between the local and central reviews in the ORR.

### 3.4. Secondary Efficacy Results

In the post-taxane population, four deaths (33.3%) were reported. The median OS was 14.42 months (95% CI: 10.35, not estimable). The Kaplan–Meier-estimated OS rates at 6 and 12 months were 90.9% (95% CI: 50.8, 98.7) and 80.8% (95% CI: 42.3, 94.9), respectively. The median duration from the first administration of ^177^Lu-PSMA-617 to the data cut-off date was 13.13 months (range: 10.0–21.3 months).

In the pre-taxane population, four deaths (22.2%) were reported. The median OS was 12.94 months (95% CI: 8.77, NE). The median should be interpreted with caution due to the small number of patients at risk and limited follow-up time at the timing of data cut-off. The Kaplan–Meier-estimated OS rates at 6 and 12 months were 94.1% (95% CI: 65.0, 99.1) and 82.4% (95% CI: 42.6, 95.7), respectively. The median duration from the first administration of ^177^Lu-PSMA-617 to the data cut-off date was 9.10 months (range: 5.4–15.5 months) (Table 3). Other secondary efficacy results are presented in Table 3 and detailed in the Supplementary Materials.

### 3.5. Patient-Reported Outcomes

The patient-reported outcome data were available for 11 out of 12 in the post-taxane population and 17 out of 18 in the pre-taxane population and are presented in Table S3. There was no notable clinical deterioration in the Functional Assessment of Cancer Therapy—Prostate (FACT-P) score from baseline to the end of treatment in both pre-and post-taxane groups.

### 3.6. Biochemical Response: Percentage Change in PSA and PSA50 Response

In the post-taxane population, the best percentage changes from baseline in mean (SD) and median PSA decreases were 27.54% (51.37) and 34.31%, respectively. PSA50 response occurred in four patients (33.3%; 95% CI: 9.9, 65.1). In the pre-taxane population, the best percentage changes from baseline in mean (SD) and median PSA decreases were 53.52% (61.985) and 85.32%, respectively. PSA50 response occurred in 10 patients (55.6%; 95% CI: 30.8, 78.5). PSA decrease was observed in 66.7% and 77.8% of the post- and pre-taxane population, respectively (Supplementary Table S1; Figure 2B).

### 3.7. Adverse Events

Overall, 93.3% of patients experienced at least one AE in the ^177^Lu-PSMA-617 SAS (91.7% and 94.4% of patients in the post- and pre-taxane populations, respectively). The most frequently reported AEs were constipation (53.3%), decreased appetite (26.7%), decreased platelet count (23.3%), anemia (20.0%), and nausea (20.0%). The most common AEs related to ^177^Lu-PSMA-617 were decreased platelet count and constipation (20.0% each), anemia (16.7%), and dry mouth and malaise (13.3% each) (Table 4). Grade 3 treatment-related events were rare, with decreased platelet count, decreased neutrophil count, lymphadenopathy, and abnormal liver function each reported in 3.3% of patients. Neither fatal AEs nor on-treatment deaths were reported. Serious AEs were observed in four patients (13.3%) and study treatment-related AEs in 20 patients (66.7%). One patient in the post-taxane group discontinued the study treatment due to an AE (grade 2 platelet count decreased) (Supplementary Table S2).

## 4. Discussion

This Phase 2 trial of targeted radioligand therapy aimed to assess the effect of ^177^Lu-PSMA-617 in patients with advanced mCRPC for the first time in Japan, mirroring clinical practice conditions in Japan, in the absence of potential cofounding effects of any new anti-cancer treatment therapies. The results of this study complement the pivotal VISION and PSMAfore trials, which assessed ^177^Lu-PSMA-617 in patients with mCRPC in post- and pre-taxane settings, respectively. This study offers valuable additional insights into the efficacy and safety of ^177^Lu-PSMA-617 in Japanese patients, a population that was underrepresented in both the VISION and PSMAfore trials.

This study intends to serve as an extension of the two pivotal trials, adopting a single-arm design to evaluate the efficacy of ^177^Lu-PSMA-617 in Japanese patients. Notably, this is the first prospective study to assess ^177^Lu-PSMA-617 in Japan, with ORR designated as the primary endpoint. The threshold for ORR was predefined based on the SoC efficacy observed in the post- and pre-taxane populations, ensuring relevance to existing treatment paradigms.

As specified in the inclusion criteria, all patients in the post-taxane arm received prior treatment with ARPI and taxane with the most common being abiraterone and docetaxel, respectively. All patients in the pre-taxane arm received prior ARPI but not taxane, with enzalutamide being the most commonly used ARPI. The treatment histories showed no significant difference between this trial and pivotal studies VISION and PSMAfore.

The study met its primary endpoint of ORR and satisfied the pre-specified threshold in both the post-taxane and pre-taxane populations. Multiple pre-planned supplementary analyses of the ORR also demonstrated robustness and consistency of these results. Therapies that achieve tumor shrinkage often have a reasonable likelihood of subsequently improving OS and other time-to-event outcomes. However, the small sample size, the inclusion of Japanese population limited to patients with at least one measurable lesion as per the PCWG3-modified RECIST v1.1, potentially representing more advanced disease, and differences in patient characteristics compared to the VISION and PSMAfore studies are important limitations to consider.

Any degree of tumor shrinkage of 41.7% and 61.1% in the post- and pre-taxane population, respectively, demonstrates the anti-tumor activity of ^177^Lu-PSMA-617. Secondary efficacy outcomes, including OS, rPFS, ORR per central radiology review, DCR, DOR, time to symptomatic skeletal events (TTSSE), and PFS also support the efficacy of ^177^Lu-PSMA-617 in Japanese patients with progressive mCRPC.

The median OS in post-taxane patients in the current study was 14.42 months, comparable to 15.3 months reported in the VISION study. For pre-taxane patients, the median OS was 12.94 months, compared to 23.66 months observed in PSMAfore study. The shorter follow-up duration with limited OS events for pre-taxane patients in the current study may have contributed to this large difference in median OS compared to PSMAfore study, highlighting the importance of longer follow-up periods in accurately estimating OS. Additionally, the median OS should be interpreted with caution due to the small number of patients at risk for both the post-taxane and pre-taxane populations.

The PSA50 response was 33.3% and 55.6% in the post- and pre-taxane populations, respectively, which is in line with the pivotal VISION (46%) and PSMAfore (51%) studies. PSA decrease was observed in 66.7% and 77.8% of the post- and pre-taxane populations, respectively. No meaningful changes were observed in clinical laboratory values including liver enzymes, vital signs, or ECG parameters.

The severity of pain and the impact of pain on daily functions were assessed using the Brief Pain Inventory—Short Form (BPI-SF), while 5-level EQ-5D (EQ-5D-5L) and FACT-P questionnaires were used to assess HRQOL. Changes in FACT-P total scores and EQ-5D-5L scores from baseline to the end of the ^177^Lu-PSMA-617 treatment were reported for both the post- and pre-taxane mCRPC Japanese patients. These changes were consistent across all subscales and domains. The findings substantiate that the treatment with ^177^Lu-PSMA-617 supported maintaining HRQOL, as reflected in the consistent results across the various scales.

Although patients in the post-taxane population received ^177^Lu-PSMA-617 plus SoC and the patients in the pre-taxane population received ^177^Lu-PSMA-617 alone, similar proportions in the incidence of AEs were observed between the two populations. Treatment with ^177^Lu-PSMA-617 demonstrated a low incidence of AEs, aligning with findings from pivotal trials. The duration of exposure to ^177^Lu-PSMA-617 was slightly longer in the pre-taxane population compared to post-taxane population, with approximately 40% of patients in both groups receiving a similar median dose intensity and approximately six infusions. No on-treatment deaths or AEs with fatal outcomes were reported. The safety profile of ^177^Lu-PSMA-617 remained consistent, showing no new or additional safety signals up to the data cut-off date, and the treatment was well tolerated with a manageable safety profile in both post- and pre-taxane settings among Japanese patients with progressive PSMA-positive mCRPC. In the pre-taxane group, the most frequently reported AEs (all grades) included constipation (38.9%), decreased appetite (22.2%), nausea (16.7%), anemia (16.7%), dry mouth (11.1%), vomiting (11.1%), and arthralgia (0%). These were comparatively less frequent than those reported in the PSMAfore ^177^Lu-PSMA-617 arm. In the post-taxane cohort, the most common AEs were dry mouth (25.0%), nausea (25.0%), anemia (25.0%), constipation (75.0%), decreased appetite (33.3%), vomiting (25.0%), and arthralgia (25.0%). These were also generally less frequent compared to the VISION trial, except for constipation, decreased appetite, and vomiting, which appeared more frequently in the current study.

These findings align with the positive outcomes observed in the VISION and PSMAfore trials, which have demonstrated prolonged rPFS, along with a favorable safety profile for ^177^Lu-PSMA-617 in patients with mCRPC. They support clinical decision-making in Japanese patients, particularly in patient selection and treatment sequencing. PSMA PET imaging plays a valuable role in identifying individuals most likely to benefit from ^177^Lu-PSMA-617. In addition to post-taxane patients, those who are not candidates for, or prefer to delay, taxane-based chemotherapy may find ^177^Lu-PSMA-617 to be a well-tolerated alternative that helps preserve QOL. Its favorable safety profile and demonstrated efficacy support its consideration earlier in the disease continuum.

Several limitations of this study should be acknowledged. First, the single-arm design precludes direct comparisons with other treatment modalities. The relatively small sample size limits the statistical power, particularly for survival outcomes and may contribute to variability in median OS estimates. Moreover, OS follow-up was not mature at the time of data cut-off, further limiting interpretation of long-term outcomes. The potential for selection bias may affect the generalizability of the findings. These limitations should be considered when interpreting the outcomes of this study.

## 5. Conclusions

With sustained tumor responses and clinically meaningful results, the current study expanded the evidence base for the efficacy and safety profile of ^177^Lu-PSMA-617 in Japanese patients with progressive mCRPC. Together, the results support ^177^Lu-PSMA-617 as an effective non-chemotherapy treatment option with a manageable safety profile for Japanese patients with progressive PSMA-positive mCRPC, demonstrating efficacy in both post- and pre-taxane populations.

## Figures and Tables

**Table 1:** Demographics and baseline characteristics ^a^.

	^177^Lu-PSMA-617 Post-Taxane Part	^177^Lu-PSMA-617 Pre-Taxane Part	^177^Lu-PSMA-617 All
	N = 12	N = 18	N = 30
**Age (years)**			
Median (range)	69.5 (50–77)	73.0 (60–82)	72.0 (50–82)
**ECOG performance status, n (%)**			
0	11 (91.7)	14 (77.8)	25 (83.3)
1	1 (8.3)	4 (22.2)	5 (16.7)
**Stage at initial diagnosis, n (%)**			
II	2 (16.7)	0	2 (6.7)
III	2 (16.7)	3 (16.7)	5 (16.7)
IV	8 (66.7)	15 (83.3)	23 (76.7)
**Metastatic sites, n (%)**			
Bone	10 (83.3)	14 (77.8)	24 (80.0)
Soft tissue	7 (58.3)	13 (72.2)	20 (66.7)
Visceral	6 (50.0)	4 (22.2)	10 (33.3)
Other	5 (41.7)	3 (16.7)	8 (26.7)
Lymph nodes	4 (33.3)	9 (50.0)	13 (43.3)
Lung or liver	2 (16.7)	2 (11.1)	4 (13.3)
Lung	1 (8.3)	2 (11.1)	3 (10.0)
**Baseline PSA (ug/L)**			
Mean (SD)	86.8 (78.1)	54.5 (94.6)	67.4 (88.5)
**Prior treatment, n (%)**			
**ARPI**			
Enzalutamide	8 (66.7)	10 (55.6)	18 (60.0)
Abiraterone acetate + Abiraterone	9 (75.0)	6 (33.3)	15 (50.0)
Apalutamide	2 (16.7)	3 (16.7)	5 (16.7)
Darolutamide	2 (16.7)	1 (5.6)	3 (10.0)
**Taxane**			
Docetaxel	12 (100.0)	0	12 (40.0)
Cabazitaxel acetone + Cabazitaxel	9 (75.0)	0	9 (30.0)

^a^ The baseline data included here are the values on or before the ^177^Lu-PSMA-617 treatment start date. ^177^Lu, lutetium-177; ARPI, androgen receptor pathway inhibitor; ECOG, Eastern Cooperative Oncology Group; and PSMA, prostate-specific membrane antigen.

**Table 2:** Efficacy of ^177^Lu-PSMA-617 in post-taxane and pre-taxane patients with measurable disease at baseline.

	Local Review (Primary Endpoint)		Central Review (Secondary Endpoint)	
Response	^177^Lu-PSMA-617 Post-Taxane Part N = 12	^177^Lu-PSMA-617 Pre-Taxane Part N = 18	^177^Lu-PSMA-617 Post-Taxane Part N = 12	^177^Lu-PSMA-617 Pre-Taxane Part N = 18
**Patients with measurable disease at baseline**	12 (100)	18 (100)	6 (50)	11 (61)
**Best overall response, n (%)**				
CR	0	4 (22.2)	1 (16.7)	3 (27.3)
PR	3 (25.0)	2 (11.1)	0	1 (9.1)
SD	8 (66.7)	9 (50.0)	3 (50.0)	4 (36.4)
**ORR: CR + PR, n (%)**	3 (25.0)	6 (33.3)	1 (16.7)	4 (36.4)
90% CI	(7.2, 52.7)	(15.6, 55.4)	(0.9, 58.2)	(13.5, 65.0)
95% CI	(5.5, 57.2)	(13.3, 59.0)	(0.4, 64.1)	(10.9, 69.2)
**DCR: CR + PR + SD +** **Non-CR/Non-PD, n (%)**	11 (91.7)	15 (83.3)	4 (66.7)	8 (72.7)
95% CI	(61.5, 99.8)	(58.6, 96.4)	(22.3, 95.7)	(39.0, 94.0)

^177^Lu, lutetium-177; CI, confidence interval; CR, complete response; DCR, disease control rate; ORR, overall response rate; PD, progressive disease; PR, partial response; PSMA, prostate-specific membrane antigen; and SD, stable disease.

**Table 3:** Secondary endpoints.

Secondary Endpoints		^177^Lu-PSMA-617 Post-Taxane Part N = 12	^177^Lu-PSMA-617 Pre-Taxane Part N = 18
**OS**	Number of events, n	4	4
	Median OS, ^a^ months (95% CI)	14.42 (10.35, NE)	12.94 (8.77, NE)
	Median follow-up, months	11.02	8.33
	Median study duration, months (range) ^b^	13.13 (10.0–21.3)	9.10 (5.4–15.5)
**rPFS**	Number of events, n	9	8
	Median rPFS, months (95% CI)	3.71 (1.87, 11.07)	12.25 (3.65, NE)
	Median follow-up, months	3.71	5.6
**TTSSE**	Number of events, n	1	3
	KM estimated EFR at 12 months, % (95% CI)	91.7 (53.9, 98.8)	NE
	Median TTSSE, months	NE	NE
**PFS**	Number of events, n	10	9
	Median PFS, months (95% CI)	3.71 (1.87, 8.51)	5.59 (3.25, NE)
	EFR at 6 months, % (95% CI)	33.3 (10.3, 58.8)	48.6 (24.1, 69.3)
**DOR**	Number of patients with confirmed CR/PR, n	3	6
	Median DOR, months (95% CI)	NE (NE, NE)	10.41 (NE, NE)
	KM estimates at 12 months, % (95% CI)	NE (NE, NE)	0.0 (NE, NE)

^a^ The median should be interpreted with caution due to the small number of patients at risk; ^b^ The median study duration between the first administration of ^177^Lu-PSMA-617 and data cut-off date. ^177^Lu, lutetium-177; CI, confidence interval; CR, complete response; DOR, duration of response; EFR, event-free rate; KM, Kaplan–Meier; NE, not estimable; OS, overall survival; PFS, progression-free survival; PR, partial response; PSMA, prostate-specific membrane antigen; rPFS, radiographic progression-free survival; and TTSSE, time to symptomatic skeletal event.

**Table 4:** AEs occurring in ≥10% in either of the treatment arms.

AEs ^a^, n (%)	All Grades n (%)			Grade ≥ 3 ^b^ n (%)		
	^177^Lu-PSMA-617 Post-Taxane Part N = 12	^177^Lu-PSMA-617 Pre-Taxane Part N = 18	^177^Lu-PSMA-617 All N = 30	^177^Lu-PSMA-617 Post-Taxane Part N = 12	^177^Lu-PSMA-617 Pre-Taxane Part N = 18	^177^Lu-PSMA-617 All N = 30
**Number of patients with ≥1 event**	11 (91.7)	17 (94.4)	28 (93.3)	1 (8.3)	5 (27.8)	6 (20.0)
**Constipation**	9 (75.0)	7 (38.9)	16 (53.3)	0	0	0
**Decreased appetite**	4 (33.3)	4 (22.2)	8 (26.7)	0	0	0
**Platelet count decreased**	2 (16.7)	5 (27.8)	7 (23.3)	0	2 (11.1)	2 (6.7)
**Anemia**	3 (25.0)	3 (16.7)	6 (20.0)	0	1 (5.6)	1 (3.3)
**Nausea**	3 (25.0)	3 (16.7)	6 (20.0)	0	0	0
**Dry mouth**	3 (25.0)	2 (11.1)	5 (16.7)	0	0	0
**Malaise**	2 (16.7)	3 (16.7)	5 (16.7)	0	0	0
**Edema peripheral**	3 (25.0)	2 (11.1)	5 (16.7)	0	0	0
**Vomiting**	3 (25.0)	2 (11.1)	5 (16.7)	0	0	0
**Dysgeusia**	1 (8.3)	3 (16.7)	4 (13.3)	0	0	0
**Arthralgia**	3 (25.0)	0	3 (10.0)	0	0	0
**Dental caries**	2 (16.7)	1 (5.6)	3 (10.0)	0	0	0
**Dysuria**	1 (8.3)	2 (11.1)	3 (10.0)	0	0	0
**Weight decreased**	1 (8.3)	2 (11.1)	3 (10.0)	0	0	0
**Dry eye**	0	2 (11.1)	2 (6.7)	0	0	0
**Leukopenia**	0	2 (11.1)	2 (6.7)	0	0	0
**Insomnia**	2 (16.7)	0	2 (6.7)	0	0	0
**Thrombocytopenia**	0	2 (11.1)	2 (6.7)	0	0	0

^a^ Patients with multiple grades for an AE are only counted under the maximum grade. Preferred terms are sorted by descending frequency in the “All” and “All grades” column; ^b^ ≥Grade 3 AEs are presented only for events with an all-grade incidence of ≥10% in either treatment arm; not all ≥Grade 3 AEs are shown. AEs that occurred from the first administration date of ^177^Lu-PSMA-617 to the last ^177^Lu-PSMA-617 administration date + 41 days or Disposition Event Date on Treatment Disposition + 30 days (or last SoC date + 30 days for post-taxane population), whichever is later. ^177^Lu, lutetium-177; AE, adverse event; SoC, standard of care; and PSMA, prostate-specific membrane antigen.

## Data Availability

Novartis is committed to sharing, with qualified external researchers, access to patient-level data and supporting clinical documents from eligible studies. These requests are reviewed and approved by an independent review panel based on the scientific merit. All data provided are anonymized to respect the privacy of patients who have participated in the trial in line with applicable laws and regulations. This trial data availability is according to the criteria and process described on www.clinicalstudydatarequest.com.

## Supplementary Materials

The following supporting information can be downloaded at: https://www.mdpi.com/article/10.3390/cancers17142351/s1, Table S1: Biochemical response; Table S2: Overview of AEs; and Table S3: Patient-reported outcomes.
