# Supplementary material for: [177Lu]Lu-PSMA-617 in Patients with Progressive PSMA+ mCRPC Treated With or Without Prior Taxane-Based Chemotherapy: A Phase 2, Open-Label, Single-Arm Trial in Japan
Source: Cancers (Basel). 2025 Jul 15;17(14):2351. doi: 10.3390/cancers17142351 (PMC12293797; doi:10.3390/cancers17142351)
Supplement: Supplementary file 1 [file cancers-17-02351-s001.zip › cancers-3690166-supplementary.pdf]

Supplementary Materials

Additional details of study design:

Patients enrolled in Part 1 were also considered in the post-taxane population (Part 2) or pre-taxane population (Part 3) for efficacy and safety assessment, depending on their pre-/post-taxane setting. The primary analysis was conducted using the data with a cut-off date of 8 December 2023 and included Part 1 to Part 3. This presents the results of the primary analysis, and Part 4 is not included.

Methods

Key Inclusion Criteria:

- ECOG performance status for the post- and pre-taxane population was 0 to 2 and 0 to 1, respectively;
- Patients must have a previous histological, pathological, and/or cytological confirmation of prostate cancer;
- Patients must have a positive ^{68}Ga-PSMA-11 PET/CT scan before enrollment to the ^{177}Lu-PSMA-617 treatment period;
- Patients must have a castrate level of serum/plasma testosterone <50 ng/dL or <1.7 nmol/L;
- Post-taxane population: patients must have received at least one ARPI in either a hormone-sensitive/castrate-resistant or non-metastatic/metastatic prostate cancer setting;
- Pre-taxane population: patients must have progressed only once on a prior second-generation ARPI and be a candidate for a change in ARPI as assessed by the treating physician;
- Post-taxane population: Patients must have been previously treated with at least one, but no more than two prior taxane regimens. If a participant has received only one taxane regimen, the participant is eligible if the participant's physician deems him unsuitable to receive a second taxane regimen;
- Patients must have progressive mCRPC based on at least one of the following criteria namely, serum PSA progression, soft-tissue progression, or progression of bone disease;
- Patients must have at least one measurable lesion per the PCWG3-modified RECIST v1.1 on CT or MRI.

Key Exclusion Criteria:

- Previous treatment with certain radiopharmaceuticals within 6 months of enrollment;
- Post-taxane population: any systemic anti-cancer therapy within 28 days prior to enrollment;
- Pre-taxane population: prior treatment with a PARP inhibitor, cytotoxic chemotherapy for prostate cancer, immunotherapy, or biological therapy;
- Known hypersensitivity to the components of ^{177}Lu-PSMA-617, ^{68}Ga-PSMA-11, or their excipients;
- Concurrent cytotoxic chemotherapy, immunotherapy, radioligand therapy, PARP inhibitors, biological treatment, AKT inhibitors, or investigational therapy;
- History of CNS metastases unless stable, asymptomatic, and not receiving corticosteroids;

- Symptomatic cord compression or findings indicative of impending cord compression.

Statistical analysis

The primary analysis included patients from Parts 1 to 3 of the study. Statistical Analysis System version 9.4 or later was used to perform all data analyses. The full analysis set included all patients who received at least one dose of ^{68}Ga-PSMA-11, regardless of administration of ^{177}Lu-PSMA-617. The primary analysis set was the primary population for efficacy data and included all the patients who were PSMA+ and received at least one dose of ^{177}Lu-PSMA-617. There were two safety analysis sets (SASs), including the PSMA-11 SAS, having all patients who received at least one dose of ^{68}Ga-PSMA-11 and the PSMA-617 SAS having all patients who were enrolled and received at least one dose of ^{177}Lu-PSMA-617.

All demographic and baseline characteristic data were summarized overall and by population. Categorical data were summarized as frequency counts and percentages, and continuous data were summarized by descriptive statistics. Dose-limiting toxicities (DLTs) were assessed based on the incidence of DLTs among evaluable patients. For the primary endpoint (ORR) analysis, the responder was defined as having a CR or PR as BOR confirmed by a second (consecutive) tumor assessment ≥4 weeks later, as per the PCWG3-modified RECIST v1.1. A one-sided exact binomial test was performed for the patients with measurable disease at baseline in the post-taxane population (Part 2) and the pre-taxane population (Part 3) of the primary analysis set. In Part 2, the null hypothesis was ORR ≤5% and the alternative hypothesis was ORR >5%. If the null hypothesis was rejected at a one-sided α level of 0.05 (i.e., the lower bound of the two-sided 90% confidence interval [CI] of ORR is >5%), then the study had to conclude on a treatment effect of ^{177}Lu-PSMA-617 plus SoC. In Part 3, the null hypothesis was ORR ≤12% and the alternative hypothesis was ORR >12%. If the null hypothesis was rejected at a one-sided α level of 0.05 (i.e., the lower bound of the two-sided 90% CI of ORR is > 12%), then the study was to conclude on a treatment effect of ^{177}Lu-PSMA-617. In both Parts 2 and 3, a point estimate of the confirmed ORR based on local review was to be presented with a 90% CI and, additionally, a 95% CI.

The secondary endpoints, OS, rPFS, time to SSE, and PFS, were estimated using the Kaplan–Meier analyses, and median, 25th percentile and 75th percentile, and associated 95% CIs were presented. PSA response rate and ORR by central review were calculated and presented with 95% CIs. Descriptive statistics were used to summarize the original score values and the change from baseline, and at various assessment timepoints in the EQ-5D-5L, FACT-P, and BPI-SF scales. AEs were summarized by the number and percentage of patients having at least one AE in each primary system organ class (SOC) and for each preferred term (PT) using MedDRA coding.

Secondary Efficacy Results

In the post-taxane population, four deaths (33.3%) were reported. The median OS was 14.42 months (95% CI: 10.35, NE). The Kaplan–Meier-estimated OS rates at 6 and 12 months were 90.9% (95% CI: 50.8, 98.7) and 80.8% (95% CI: 42.3, 94.9), respectively. The median follow-up time for OS

was 11.02 months, with a median duration from the first administration of ^{177}Lu-PSMA-617 to the data cut-off date of 13.13 months (range: 10.0-21.3 months).

In the pre-taxane population, four deaths (22.2%) were reported. The median OS was 12.94 months (95% CI: 8.77, NE). The Kaplan–Meier-estimated OS rates at 6 and 12 months were 94.1% (95% CI: 65.0, 99.1) and 82.4% (95% CI: 42.6, 95.7), respectively. The median follow-up time for OS was 8.33 months, with a median duration from the first administration of ^{177}Lu-PSMA-617 to the data cut-off date of 9.10 months (range: 5.4-15.5 months) (**Table 3**).

rPFS

In the post-taxane population, all 12 patients had measurable disease at baseline, with 9 (75.0%) experiencing radiographic progression events. No deaths were reported. The median rPFS was 3.71 months (95% CI: 1.87, 11.07). The Kaplan–Meier-estimated event-free rates at 6 and 12 months were 41.7% (95% CI: 15.2, 66.5) and 20.8% (95% CI: 3.5, 47.9), respectively. Based on central radiology review, 50.0% (6/12) had measurable disease at baseline, with a median rPFS of 11.07 months (95% CI: 1.87, NE). The Kaplan–Meier-estimated event-free rates were 66.7% (95% CI: 19.5, 90.4) at 6 months and 33.3% (95% CI: 1.4, 75.5) at 12 months. The median follow-up time for rPFS was 3.71 months.

In the pre-taxane population, all 18 patients had measurable disease at baseline, with 8 (44.4%) experiencing radiographic progression events. No deaths were reported. The median rPFS was 12.25 months (95% CI: 3.65, NE). The Kaplan–Meier-estimated event-free rates at 6 and 12 months were both 59.1% (95% CI: 32.5, 78.2). Based on central radiology review, 61.1% (11/18) had measurable disease at baseline, with the median rPFS not estimable. The Kaplan–Meier-estimated event-free rate was 63.6% (95% CI: 29.7, 84.5) at 6 months. The median follow-up time for rPFS was 5.60 months.

DCR

In the post-taxane population, the DCR based on local radiology review was 91.7% (95% CI: 61.5, 99.8), with PR in three patients (25.0%) and (SD in eight patients (66.7%). No CR or non-CR/non-PD cases were reported. Based on central radiology review, the DCR was 66.7% (95% CI: 22.3, 95.7), with CR in one patient (16.7%) and SD in 3 patients (50.0%).

In the pre-taxane population, the DCR based on local radiology review was 83.3% (95% CI: 58.6, 96.4), with CR in four patients (22.2%), PR in two patients (11.1%), and SD in nine patients (50.0%). Based on central radiology review, the DCR was 72.7% (95% CI: 39.0, 94.0), with CR in three patients (27.3%), PR in one patient (9.1%), and SD in four patients (36.4%).

DOR

In the post-taxane population, three patients (25.0%) had confirmed CR/PR, with none experiencing radiographic progression or death. The Kaplan–Meier-estimated event-free rate at 6 months was 100.0% (95% CI: 100.0, 100.0), and it was not estimable at 9 months or later.

In the pre-taxane population, six patients (33.3%) had confirmed CR/PR. One patient (16.7%) experienced a radiographic progression event, but no deaths were reported. The median DOR was 10.41 months (95% CI: NE, NE). The other five patients (83.3%) were censored and ongoing without events. The Kaplan–Meier-estimated event-free rate at 9 months was 100.0% (95% CI: 100.0, 100.0), and it was not estimable at 12 months.

TTSSE

In the post-taxane population, only one patient (8.3%) experienced a symptomatic skeletal event, making the median TTSSE not estimable. The Kaplan–Meier-estimated event-free rate for TTSSE at 12 months was 91.7% (95% CI: 53.9, 98.8), and it was not estimable at 15 months. In the pre-taxane population, three patients (16.7%) experienced symptomatic skeletal events, with two requiring radiation therapy for bone pain and one experiencing spinal cord compression. The median TTSSE was not estimable. The Kaplan–Meier-estimated event-free rate for TTSSE at 9 months was 81.1% (95% CI: 51.9, 93.5), and it was not estimable at 12 months.

PFS

In the post-taxane population, all 12 patients (100.0%) had measurable disease at baseline, with 10 patients (83.3%) experiencing progression events. No deaths were reported. The median PFS was 3.71 months (95% CI: 1.87, 8.51). The Kaplan–Meier-estimated event-free rate at 6 months was 33.3% (95% CI: 10.3, 58.8) and was not estimable at 12 months. In the pre-taxane population, all 18 patients (100.0%) had measurable disease at baseline, with 9 patients (50.0%) experiencing progression events. No deaths were reported. The median PFS was 5.59 months (95% CI: 3.25, NE). The Kaplan–Meier-estimated event-free rate at 6 months was 48.6% (95% CI: 24.1, 69.3) and was not estimable at 12 months.

Table S1: Biochemical response

**Best % decrease**	**PSA levels, μg/L**		**LDH levels, U/L**		**ALP levels, U/L**	
	**Post-taxane N=12**	**Pre-taxane N=18**	**Post-taxane N=12**	**Pre-taxane N=18**	**Post-taxane N=12**	**Pre-taxane N=18**
**Baseline, mean (SD)**	86.82 (78.12)	54.49 (94.64)	352.3 (238.04)	226.1 (72.11)	85.3 (36.82)	104.7 (57.10)
**Baseline, median**	64.13	26.34	260.0	210.5	79.5	88.5
**Best % change from baseline, mean (SD)**	−27.54 (51.37)	−53.52 (61.99)	−21.29 (14.636)	−17.15 (10.201)	−13.99 (20.356)	−20.40 (20.332)
**Best % change from baseline, median**	−34.31	−85.32	−23.54	−16.38	−10.05	−9.95
**PSA50 response rate, % (95% CI)**	33.3 (9.9, 65.1)	55.6 (30.8, 78.5)				

ALP, alkaline phosphatase; CI, confidence interval; LDH, lactate dehydrogenase; PSA, prostate-specific antigen; and SD, standard deviation.

Table S2: Overview of AEs

Category	^{177}Lu-PSMA-617 Post-taxane part N=12		^{177}Lu-PSMA-617 Pre-taxane part N=18		^{177}Lu-PSMA-617 All N=30	
	All grades n (%)	Grade ≥3 n (%)	All grades n (%)	Grade ≥3 n (%)	All grades n (%)	Grade ≥3 n (%)
**AEs**	11 (91.7)	1 (8.3)	17 (94.4)	5 (27.8)	28 (93.3)	6 (20.0)
Treatment-related	8 (66.7)	1 (8.3)	12 (66.7)	2 (11.1)	20 (66.7)	3 (10.0)
^{177}Lu-PSMA-617 related	8 (66.7)	1 (8.3)	12 (66.7)	2 (11.1)	20 (66.7)	3 (10.0)
**SAEs**	2 (16.7)	1 (8.3)	2 (11.1)	2 (11.1)	4 (13.3)	3 (10.0)
Treatment-related	1 (8.3)	1 (8.3)	0	0	1 (3.3)	1 (3.3)
^{177}Lu-PSMA-617 related	1 (8.3)	1 (8.3)	0	0	1 (3.3)	1 (3.3)
**AEs leading to study treatment discontinuation**	1 (8.3)	0	0	0	1 (3.3)	0
Treatment-related	1 (8.3)	0	0	0	1 (3.3)	0
^{177}Lu-PSMA-617 related	1 (8.3)	0	0	0	1 (3.3)	0

A patient with multiple grades for an AE is only counted under the maximum grade. AEs that occurred from the first administration date of the ^{177}Lu-PSMA-617 to the last ^{177}Lu-PSMA-617 administration date + 41 days or the disposition event date on treatment disposition + 30 days (or the last SoC date + 30 days for post-taxane population), whichever is later. ^{177}Lu, lutetium-177; AE, adverse event; BSC, best supportive care; SoC, standard of care; PSMA, prostate-specific membrane antigen; and SAE, serious adverse event.

Table S3: Patient-reported outcomes

Measure[a]	BPI-SF Pain at its Worst in the Last 24 Hours		BPI-SF Intensity Scale		BPI-SF Interference Scale		FACT-P Total Score		EQ-5D-5L Japan Utility Score		EQ-5D-5L - EQ-VAS	
	Post-taxane N=12	Pre-taxane N=18	Post-taxane N=12	Pre-taxane N=18	Post-taxane N=12	Pre-taxane N=18	Post-taxane N=12	Pre-taxane N=18	Post-taxane N=12	Pre-taxane N=18	Post-taxane N=12	Pre-taxane N=18
**n**	11	17	11	17	11	17	11	17	11	16	11	16
**Baseline mean (SD)**	2.73 (2.102)	2.82 (3.046)	2.05 (1.642)	2.35 (2.391)	1.16 (1.170)	1.77 (2.060)	107.70 (17.407)	107.13 (21.718)	0.88 (0.125)	0.84 (0.171)	78.27 (14.471)	77.25 (18.972)
**n**	10	15	10	15	10	15	10	15	10	14	10	14
**C3W1 mean (SD) change from baseline**	0.10 (2.885)	0.80 (2.883)	0.10 (2.630)	0.42 (1.782)	0.91 (2.587)	0.35 (1.076)	−2.82 (12.461)	−3.78 (11.049)	−0.03 (0.155)	0.02 (0.093)	−2.70 (19.460)	−1.57 (18.455)
**n**	4	6	4	6	4	6	4	6	4	6	4	6
**C6W1 mean (SD) change from baseline**	−1.25 (2.500)	−0.17 (0.408)	−1.50 (2.380)	0.00 (0.570)	−0.89 (1.214)	0.17 (0.660)	6.54 (24.812)	−2.31 (8.432)	−0.11 (0.251)	0.03 (0.156)	−3.50 (14.387)	6.00 (7.071)
**n**	10	13	10	13	10	13	10	13	10	12	10	12
**EoT mean (SD) change from baseline**	0.90 (2.885)	0.69 (1.548)	1.13 (2.425)	0.69 (1.374)	1.89 (1.655)	0.82 (1.702)	−17.63 (22.278)	−7.82 (15.475)	−0.16 (0.121)	-0.08 (0.275)	−7.30 (19.551)	−3.25 (21.659)

[a]Low scores may not always indicate favorable results; it depends on the actual scale. ^{177}Lu, lutetium-177; C3W1, cycle 3 week 1; C6W1, cycle 6 week 1; BPI-SF, Brief Pain Inventory—Short Form; EoT, end of treatment; EQ-5D-5L, EuroQoL-5 Dimension-5 Level; EQ-VAS, EuroQoL Visual Analog Scale; FACT-P, Functional Assessment of Cancer Therapy—Prostate; HRQoL, health-related quality of life; PSMA, prostate-specific membrane antigen; and SD, standard deviation.
